# Lacerations to Zones VIII and IX: It Is Not Just a Tendon Injury

**DOI:** 10.4061/2011/261681

**Published:** 2010-09-14

**Authors:** Charla R. Fischer, Peter Tang

**Affiliations:** Department of Orthopaedic Surgery, Columbia University Medical Center, 622 West 168th Street, PH11-1130, New York, NY 10023, USA

## Abstract

Extensor tendon injuries are widely believed to be straightforward problems that are relatively simple to manage. However, these injuries can be complex and demand a thorough understanding of anatomy to achieve the best functional outcomes. When lacerations occur in the forearm as in Zones VIII and IX injury, the repair of the extensor tendon and muscle, and posterior interosseous nerve (PIN) is often challenging. A review of the literature shows little guidance and attention for these injuries. We present four patients with injuries to Zones VIII and IX as well as a review of surgical technique, postoperative rehabilitation, and pearls that may be of benefit to those managing these injuries.

## 1. Introduction

There is a prevailing assumption that extensor tendon injuries are not difficult to manage because they all have good outcomes [1, 2]. This may be due to the fact that extension in the hand is not essential to normal function and small losses in extension are easily compensated due to redundancy built into the extensor tendon system [1]. Additionally, it may be due to the superficial location of these tendons making them more accessible than flexor tendons [3, 4]. This assumption may also explain the paucity in the literature on the long-term outcomes following extensor tendon repair [5], and on how to manage extensor tendon injuries especially in Zones VIII and IX (as classified by Verdan) [6]. We have recently treated four of these injuries: three due to assault with a machete and one due to a fall on broken glass. The machete injury is similar to the “nightstick” fracture injury as it is natural reflex is to protect one's face/head with one's forearm during an attack. The purpose of this study is to highlight that extensor tendon injuries in Zones VIII and IX are challenging due to the proximity of the musculotendinous junction, and injury to the posterior interosseous nerve (PIN). We provide a surgical technique for treating these patients.

## 2. Materials and Methods

This is a retrospective chart review of four patients who presented to our institution between July 6, 2008 and June 9, 2009 with injuries to Zones VIII and XI. Zone VIII is defined as proximal to the extensor retinaculum synovial sheaths of the extensor tendons at the wrist and includes the distal one fourth of the forearm. Zone XI is defined as the proximal three fourths of the forearm [7]. Three patients were assaulted with a machete and one fell on broken glass. These patients all underwent primary operative repair by the senior surgeon (PT). Three patients had posterior interosseous nerve lacerations in addition to multiple extensor tendon lacerations. After our institutional review board approved this study, these patients gave informed consent to participate in the study. All four cases are illustrated below. Patient results were evaluated according to Dargan's [2] method: excellent, no flexion or extension lag; good, no flexion lag, extension lag of 15 degrees or less; fair, pulp to palm distance of 2 cm or less, extension lag 15 to 45 degrees; and poor, pulp to palm distance more than 2 cm, extension lag more than 45 degrees. We did not perform statistical analysis. Furthermore, a cadaveric dissection was done to illustrate anatomy.

## 3. Case Series

### 3.1. Case Number 1

The first case is a 30-year-old right hand dominant male with a 12 cm laceration from a machete at the midportion of the forearm. On exam he could not extend his thumb or any of his fingers and could only extend his wrist in a radial direction. Radiographs show an ulna fracture from the machete. He was taken to surgery the day of the injury and was found to have all extensor tendons lacerated except for the extensor carpi radialis longus (ECRL) and 10% of the extensor carpi radialis brevis (ECRB). The main trunk and two branches of the PIN were also lacerated. The depth of the wound ended at the ulna where there was an incomplete fracture representing arrest of the machete's path by the ulna. 

 In terms of surgical technique, the wound was extended proximally and distally. The skin and subcutaneous adipose layer were elevated as flaps. Individual fascial compartments were incised for exposure. As tendons were found they were tagged in a modified Kessler fashion with 2.0 Ticron leaving the ends long for later repair. If the corresponding tendon could be found easily, this was also tagged and the sutures of the two tendon ends were clamped with a hemostat. 

 Repairs should be performed from deep to superficial so a superficial repair will not obstruct the repair of a deeper structure. In this case the PIN was the deepest structure that needed to be repaired. The nerve ends were dissected proximally and distally so that a tension free primary repair could be achieved with 8.0 nylon using 2.5x loupe magnification. Two sutures were placed for each nerve repair. Once the PIN was repaired the tendon lacerations were next addressed.

Primary repairs were done of the extensor digitorum communis (EDC) of the middle and ring fingers, the abductor pollicis longus (APL), and the extensor carpi ulnaris (ECU). Only one extensor tendon to the small finger was found and repaired. Most likely it was the extensor digiti minimi (EDM) and the patient lacked a small finger EDC. The EDC to the index was also repaired. We believed the extensor indicis proprius (EIP) tendon and muscle was uninjured since its origin was distal to the site of injury but there was no index finger extensor on exam because of the PIN had been lacerated. We believe the same applies to the extensor pollicis brevis (EPB). Because the extensor pollicis longus (EPL) was lacerated very proximal at the musculotendinous junction there was no available proximal tendon to repair. Therefore, an end to side transfer to the middle finger EDC was performed. Likewise, the index EDC repair was not robust proximally, so a side-to-side transfer was done to the ring finger EDC to supplement/replace the primary repair. 

The wound was irrigated copiously and the skin was closed with 4.0 nylon. A dressing was placed as well as a below-elbow volar splint with the hand in the intrinsic plus position including a thumb spica and the wrist in 20 degrees of extension. This patient has failed to return for followup.

### 3.2. Case Number 2

The second case is a 13-year-old left hand dominant male who sustained a laceration at the junction of the middle and proximal third of the right dorsal forearm after falling on a large piece of glass. He was unable to extend his thumb and small finger, and could only extend his wrist radially. Radiographs show a small piece of glass at the level of the injury. Intraoperatively, there were complete lacerations of the EPL, the small finger EDC, and the ECU. There was an incomplete laceration of the EDM. Also, the main trunk and two branches of the PIN were found to be transected. There was also an incomplete fracture of the ulna from the glass. 

 The EPL was injured very proximal at the musculotendinous junction so there was no tendon proper proximally. 2.0 Ticron was placed in a figures-of-eight fashion, proximally in muscle and any available fascial tissue including intramuscular septae. The EPL injury was deep to the nerve injury so this was repaired first. Next, the main trunk of the PIN which was 2 mm in diameter was repaired with 8.0 nylon. The two smaller branches were repaired with 9.0 nylon. The main trunk and one of the smaller branches was under some tension despite mobilization of the nerves proximally and distally. Thus, a type I collagen nerve wrap (NeuroMend, Stryker Orthopaedics, Mahwah, New Jersey) was placed around these two repairs. Due to the musculotendinous level of injury of the small finger EDC and EDM, multiple figure-of-eight 0.0 Ticron sutures were placed into any tendon substance available but proximally there was only muscle substance. The ECU had tendon available proximal and distal to the level of the injury so 0.0 Ticron was used in a modified Kessler fashion supplemented with a horizontal mattress.

**Figure 1:**
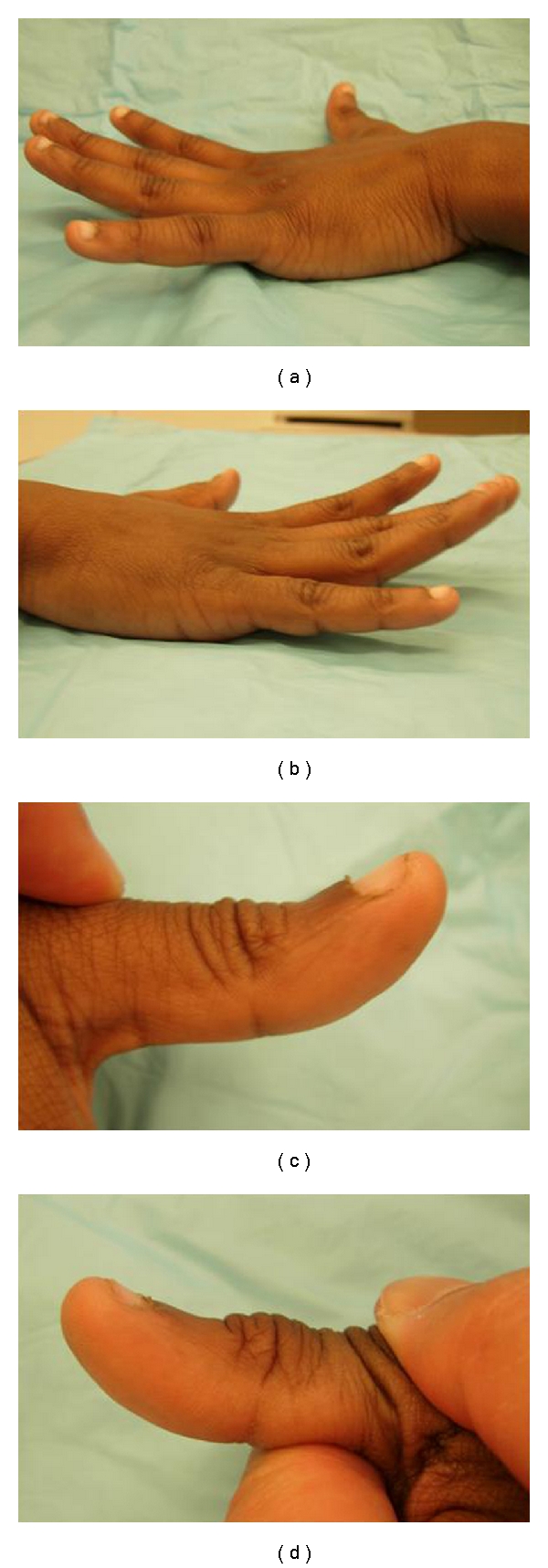
Seven months postoperative range of motion of 13-year-old right hand dominant male who sustained glass laceration to his right dorsal forearm. The range of motion of the left hand is shown compared to the right: (a) left small finger extension 20°, (b) right finger small finger extension 15°, (c) left thumb IP extension 35° with proximal phalanx extended, and (d) right thumb IP flexion 20° with proximal phalanx extended.

Postoperatively, he was placed in a splint with the wrist in 20 degrees of extension and the fingers and thumb in extension for 2 weeks. After 2 weeks he was then placed in a cast for compliance issues given his age, for 4 weeks with the wrist in 20 degrees extension and the finger and thumb MCP's in extension. Finger motion within the confines of the splint was started with occupational therapy (OT). At 6 weeks immobilization was discontinued, and full active and passive motion was started. Resistive exercises were started at 3 months. At 7 months he has regained full function and near full range of motion with a Dargan grading of good. The small finger metacarpophalangeal (MCP) joint range of motion is 15/85 degrees, compared to 20/85 degrees on his left side (Figures 1(a)–1(d)). His thumb interphalangeal joint extension is 40 degrees when the MCP is held in flexion and 20 degrees when the proximal phalanx is extended. On his left side, he is able to extend to 60 degrees and 35 degrees, respectively. He can also extend his wrist without radial deviation.

### 3.3. Case Number 3

**Figure 2:**
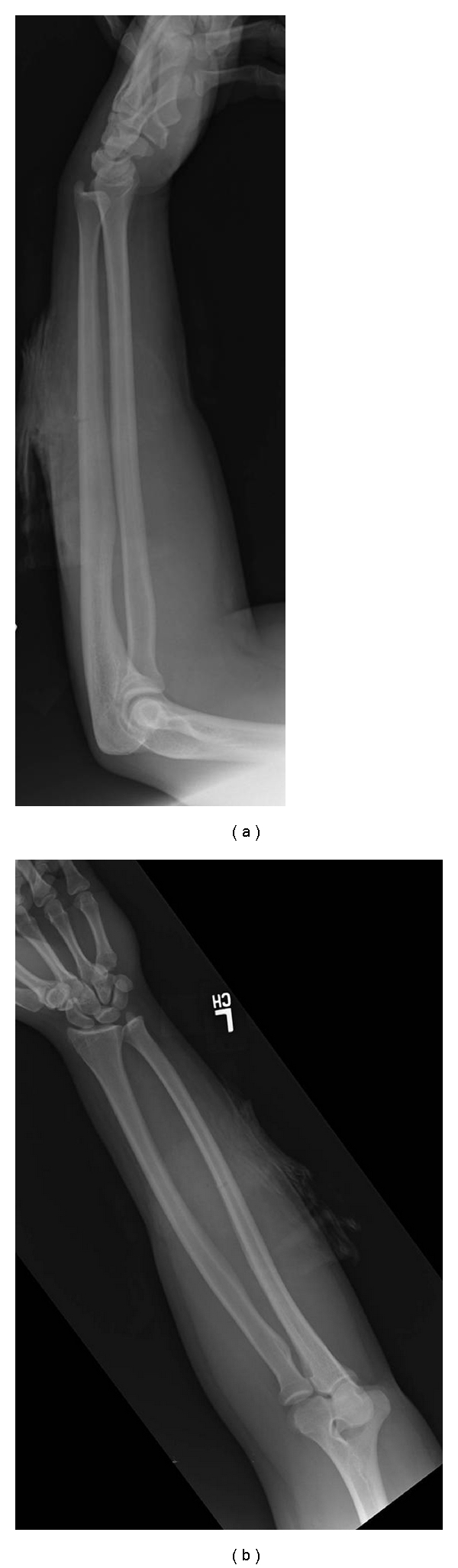
Preoperative (a) anteroposterior and (b) lateral forearm radiographs of 17-year-old right hand dominant male who was struck with a machete on his left dorsal forearm.

The third case is 17-year-old right hand dominant male who was struck with a machete in the middle of his left dorsal forearm. He sustained a 10 cm laceration that extended to the ulna. He was unable to extend his thumb, middle, ring and small fingers. He could only extend his wrist in a radial direction. Radiographs again show a fracture from the machete in the middle of the ulna (Figures 2(a) and 2(b)). He was taken to surgery within 24 hours of his injury and lacerations of the index, middle, ring, and small finger EDC, EDM, EPL and ECU were found. Also, the posterior interosseous nerve was lacerated with a 1 cm gap. Even after nerve mobilization, a tension-free primary repair could not be achieved so Neuroflex, a flexible type I collagen conduit, (Stryker Orthopaedics, Mahwah, New Jersey) was used as a conduit to bridge the gap. The nerve conduit was secured with two 9.0 nylon sutures at the proximal and distal ends. Primary tendon repair was performed using 2.0 Vicryl in a modified Kessler fashion supplemented with a horizontal mattress suture. Postoperatively, a splint was placed immobilizing his wrist in 20 deg of extension and fingers and thumb in full extension. He was lost to followup.

### 3.4. Case Number 4

**Figure 3:**
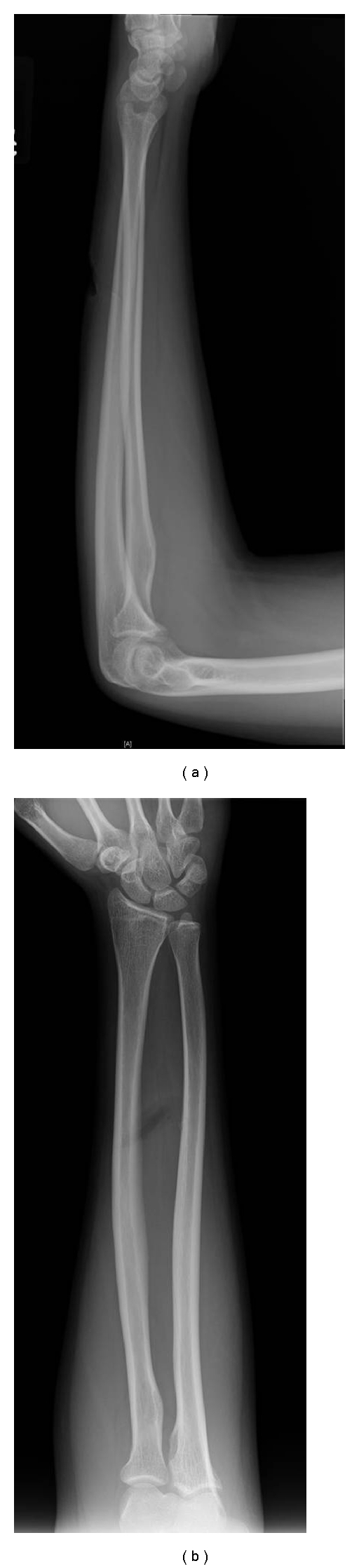
Initial (a) anteroposterior and (b) lateral films of 18-year-old right hand dominant male who sustained machete laceration to his left dorsal forearm.

**Figure 4:**
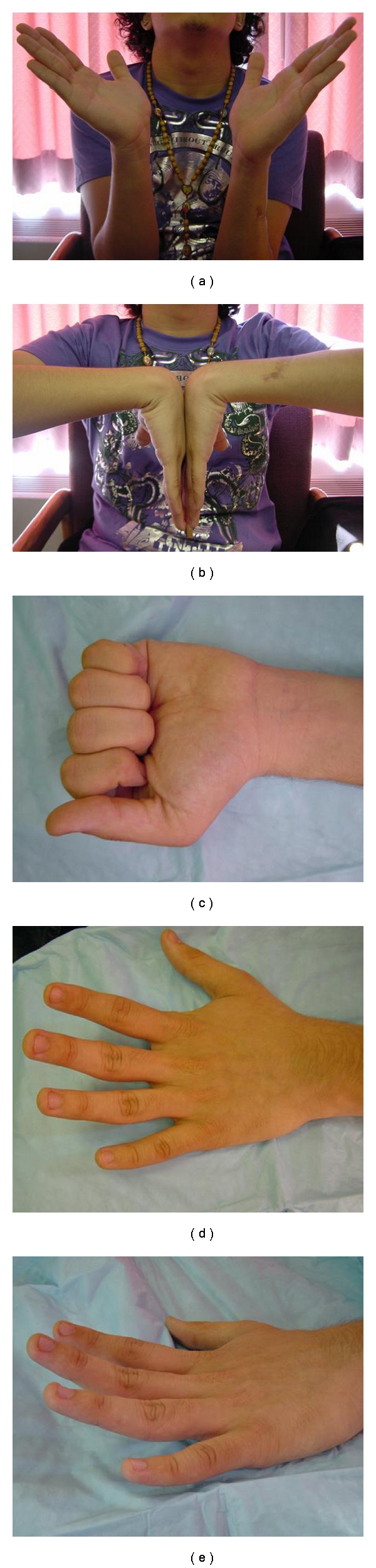
Seven months postoperative ROM of 18-year-old RHD male who sustained a laceration to his left dorsal forearm. The motion is as follows: (a) 50° wrist extension; (b) 90° wrist flexion; (c) full finger flexion into a fist; (d, e) full finger extension off table.

The fourth case is a 18-year-old right hand dominant male who was assaulted with a machete. His laceration was 3 cm in length and located at the middle third of the ulnar aspect of his left dorsal forearm. He was unable to extend his middle, ring and small fingers. He was able to extend his wrist only in a radial direction. Radiographs again show and ulna fracture from the machete (Figures 3(a) and 3(b)). Intraoperatively, his injury included lacerations of the index, middle, ring and small finger EDC, EDM, and ECU. He did not have a PIN injury. He underwent primary tendon repair with 3.0 Fiberwire (Arthrex, Naples, Florida) in a modified Kessler fashion supplemented with horizontal mattress suture. The ECU repair was further supplemented with an epitendinous repair with 5.0 nylon. His fingers and wrist were immobilized in the intrinsic plus position for 2 weeks after surgery. At the two week visit, OT fabricated a volar wrist splint that included the MCP joint's in extension and the wrist is 20 deg of extension. Therapy was started with active and passive ROM with the restriction of no resistive exercises. The splint was discontinued at 6 weeks and strengthening was started. At the most recent follow up at seven months, he had excellent results according to Dargan's classification. He has recovered full extension of his third, fourth, and fifth fingers and well as normal wrist extension (Figures 4(a)–4(e)). His only complaint was of mild stiffness with wrist extension.

## 4. Discussion

Extensor tendon injuries are assumed to be simple to manage because they all have good outcomes [1, 2], and often the least experienced surgeon handles these injuries in less than ideal settings [2]. This may explain the paucity in literature about Zone VIII and IX extensor tendon injuries which we would contend is a significant injury and is the focus of this paper. In terms of the anatomy, the dorsal forearm has a superficial and deep layer of muscles. Listing the muscles from radial to ulnar, the superficial layer consists of the ECRL, ECRB, EDC, EDM, and ECU. Again from radial to ulnar, the deep layer consists of the APL, EPB, EPL, and EIP. The PIN enters the dorsal forearm compartment by splitting the two heads of the supinator and emerging from under the Arcade of Frohse. It then travels between the superficial and deep muscle layers giving innervation to these muscles. 

Besides knowledge of the dorsal forearm anatomy, other tips to help match tendons ends include matching the contents of the proximal and distal extent of the individual fascial compartments which have also been lacerated, and evaluating the radial/ulnar and superficial/deep location of the tendons and muscle bellies. Lastly, tendons ends often retract within the muscle bellies and must be identified by dissection. 

The injury with a machete is similar in mechanism to the “nightstick” injury as it is natural reflex to protect one's face and head by blocking the object with one's dorsal forearm. In all of our cases including the one with glass there was an indentation left by the machete or glass representing the cessation of travel of the sharp object by the bone. How the forearm is presented in terms of rotation and where (proximal/distal location) the forearm is struck will dictate which and how many extensor tendon are injured. Consistently, in all our cases the ECU was injured due to its dorsal, ulnar location and being adjacent to the ulna. 

**Figure 5:**
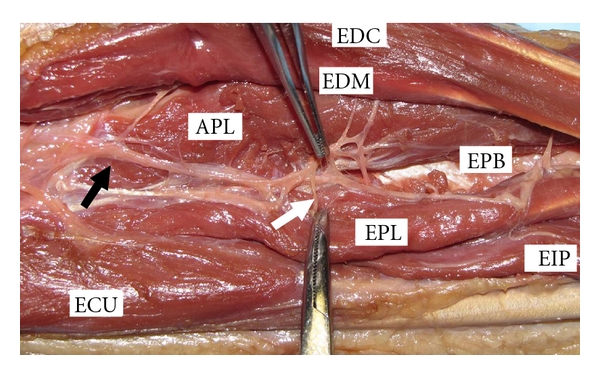
Cadaveric gross dissection of dorsal forearm. The extensor pollicis brevis (EPB), extensor digit minimi (EDM), extensor digitorum comunis (EDC), extensor carpi ulnaris (ECU), extensor pollicis longus (EPL), and extensor incidis proprius (EIP) are labeled. The black arrow indicates the posterior interosseous nerve after exiting the arcade of Frohse. The white arrow indicates a posterior interosseous nerve (PIN) branch to EPL.

**Figure 6:**
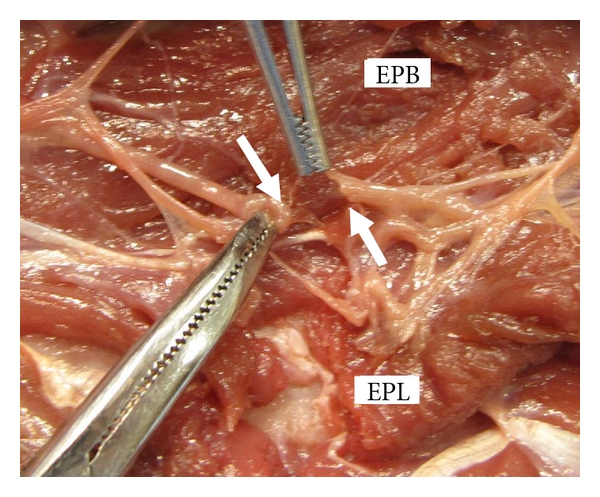
Cadaveric gross dissection with laceration injury recreated showing cut extensor carpi ulnaris (ECU), extensor pollicis longus (EPL), and white arrows indicate cut branch of posterior interosseous nerve (PIN) to extensor pollicis longus (EPL).

The close proximity of the PIN to the site of injury makes its involvement likely as illustrated in our case series with PIN involvement in three of our four cases (Figure 5). This is highlighted in case number 1 where the patient lacked EIP and EPB function not because of injury to the musculotendinous unit but because of injury to the nerve. With lacerations to Zone IX, when the APL, EPL, EPB and EIP fail to function there should be suspicion that PIN injury is the sole reason or part of the reason for dysfunction (Figure 6). This is because their origins are the most distal on the forearm of all the extensor muscles and they are the last to be innervated by the PIN [8]. The evaluation of the index finger is complicated by the fact that the both the index EDC and EIP extend it. Thus, when the index finger fails to extend, it represents dysfunction of both these tendons but for a Zone IX laceration the pattern of injury most likely consists of laceration of the index EDC musculotendinous unit and transection of the PIN to the EIP. Conversely, if the EIP is intact (hyperextension of the index finger at the MCP is possible with the other fingers held in flexion) then some of the musculotendinous unit as well as its innervation is intact. If there is any PIN injury it only involves a nonEIP branch and not the main trunk since the EIP is usually the last extensor muscle to be innervated [8]. 

Dorsal forearm lacerations are unique when compared to all upper extremity lacerations in that motor nerve involvement must be appreciated during assessment of the injury. On the dorsal side, lacerations distal to Zone VIII involve injury only to tendons and any nerve involvement would only be sensory in nature in the form of the superficial radial sensory nerve. On the volar side, lacerations to the volar aspect of the fingers involve tendon, digital sensory nerves and possibly digital arteries. Lacerations to the volar wrist can involve motor fibers of the median and ulnar nerves but except for the anterior interossesous nerve (AIN) innervation of the pronator, the innervation is not occurring at the site of injury. Injury that involves motor nerves at the level of muscle innervation as seen in extensor Zones VII and IX injuries would only occur with deep lacerations of the palm, which are relatively rare though not unheard of, with the intrinsics or with lacerations in the proximal forearm which are rare, with the flexor tendon muscles.

Because this injury pattern is confusing we devised an injury classification describing lacerations of musculotendinous units with or without innervating nerve involvement. Type I involves motor nerve injury alone and is proximal to muscle innervation. There is no injury to the musculotendinous unit but the unit is flail because of the nerve injury. Type II involves injury to the muscle substance at the site of muscle origin with (a) being a partial laceration and (b) being a complete laceration. If there is no nerve involvement and because the injury is at the site of origin whether it is partial or complete laceration, there should some proximal anchor for the musculotendinous unit to pull against when firing so some function of the unit will be preserved. Type II(n) involves the nerve. Type II(n)a theoretically will preserve some musculotendinous function as the proximally innervated muscle can fire and pull past the partial laceration and ultimately, pull the distal tendon. Type II(n)b theoretically will not preserve function as there will be no innervated muscle to pull the distal tendon. Type III involves injury to the musculotendious junction distal to the muscle origin with (a) being partial and (b) complete. In this type a partial (Type IIIa) injury will preserve some function of the musculotendinous unit, while a complete (Type IIIb) injury will not because there will be no attachment of the unit proximally. Type III(n) involves nerve injury. Partial injuries (Type III(n)a) will preserve some function while complete injuries (Type III(n)b) will not. Type IV injures involve only tendon ((a) partial laceration, (b) complete laceration and no nerve injury,

Type I: motor nerve injury alone proximal to muscle innervation, no injury to the musculotendinous unit
Type II: injury to the muscle substance at the site of muscle origin, (a) partial laceration, (b) complete laceration.
Type II(n): above plus motor (n)erve injury
Type III: injury to the musculotendious area distal to the muscle origin, (a) partial laceration, (b) complete laceration.
Type III(n): above plus motor (n)erve injury.
Type IV: tendon injury alone, no nerve injury, (a) partial laceration, (b) complete laceration.

For these injuries microsurgical instruments should be available. Not uncommonly primary repair without tension may not be possible even when the nerve ends are mobilized. In one of our cases we needed a nerve conduit to bridge a 1 cm gap despite the procedure occurring within 24 hours of injury. In another case there was some tension on two of the three repairs and a nerve wrap was utilized. Thus, it is important to have available a nerve conduit, wrap, or allograft (Avance, Axogen) and to consent the patient for possible autograft if that is the surgeon's preference. 

In terms of repair of the musculotendinous unit knowledge of the dorsal forearm anatomy is useful. Knowledge of this anatomy will aid in appropriately matching the injured extensor tendons and muscles. Tips to help match tendons ends include matching the contents of the proximal and distal extent of the individual fascial compartments which have also been lacerated. For instance, the EPB tendon is more radial to the EPL but its origin is located more distally. Another pearl is to match structures in both the radial/ulnar and superficial/deep location. Since there can be many tendons to repair, tagging the tendons in a modified Kessler fashion and clamping the suture end as is done during repair of the “spaghetti wrist” (volar wrist lacerations) will be helpful. If the matching tendon end is found, clamping the pair of sutures of the matching tendon ends will keep the repair organized. Furthermore, matching the contents of corresponding compartments will be helpful. Besides location, tendon ends can also be matched by size and the relatively lengths of each end. Repair sequence should intuitively be performed from deep to superficial whether it be tendon/muscle or nerve so that superficial structures will not obstruct the repair of deeper ones. Lastly, tendons ends often retract within the muscle bellies and must be identified by dissection. 

Another challenging aspect of Zones VIII and IX repair is the musculotendinous junction injury site. The lack of tendon proper proximally makes suture hold proximally often inadequate. Any available fascia proximally whether it be intramuscular fascial septae or even compartment fascia is used to strengthen the repair. If this fails or a weak repair is to be supplemented, one can perform side-to-side transfers. An adjacent musculotendinous unit may have more tendon proper proximally which will give more proximal hold to the repair. This technique was employed by Takami and co-workers with traumatic rupture of the extensor tendons at the musculotendinous junction where they often found a direct repair “impossible” [3]. The downside to this technique is that it would inhibit independent finger extension and created a “mass action” of finger extension. However, the design of the extensor tendon system with the common muscle belly of the EDC, the juncturae tendinum, and intertendinous fascia makes finger extension an nonindependent phenomena [9]. Independent finger extension occurs for the index and small finger because of the additional tendons to the EDC, the EIP and EDQ, respectively. A last alternative when the laceration is 50% or more of at least two muscle bellies, is to use a palmaris longus or toe extensor graft to weave into the superficial and deep epimysium and then suturing the ends to themselves in a Pulvertaft fashion [10]. 

As for postoperative protocol for Zone VIII injuries, some literature recommends static immobilization for 5 to 6 weeks with the wrist extended 45 degrees [11], while others recommend the same wrist position as well as the metacarpophalangeal (MCP) joints in 15 to 20 deg of flexion for 4 to 5 weeks [7]. Still others recommend splinting the elbow in flexion since the extensors originate from the lateral epicondyle [12]. Dynamic splinting has been advocated by some but for extensor injuries from Zone IV to VII with good results [4]. Our general rule is if we are satisfied with the strength of our repair we keep the postoperative splint on for two weeks. At the two week postop visit the sutures are removed and we place the patient in a custom-made splint with the wrist in 20 degrees of extension and the affected fingers MCP joints in 70 degrees of flexion. If the EPL tendon is involved the thumb MCP joint is placed in extension. These removable splints are kept on all the time except for bathing, therapy and home exercises. Therapy is also started at this time including active and passive range of motion with the restriction of no resistive exercises with no strenuous finger flexion and extension. However, if we are not satisfied about the strength of the repair as is often is the case with lacerations at the musculotendinous junction because of proximal suture hold, then we may statically splint them and start therapy later or place a removable splint and start therapy later. Therapy in these tenuous repair situations may be the same as in the robust repairs or a passive motion protocol may be utilized.

## References

[1] Blue  AI, Spira M, Hardy SB (1976). Repair of extensor tendon injuries of the hand. *American Journal of Surgery*.

[2] Browne EZ, Ribik CA (1989). Early dynamic splinting for extensor tendon injuries. *The Journal of Hand Surgery*.

[3] Takami H, Takahashi S, Ando M, Suzuki K (1995). Traumatic rupture of the extensor tendons at the musculotendinous junction. *The Journal of Hand Surgery*.

[4] Mason ML (1959). Primary tendon repair. *Journal of Bone & Joint Surgery*.

[5] Newport ML, Blair WF, Steyers CM (1990). Long-term results of extensor tendon repair. *The Journal of Hand Surgery*.

[6] Verdan CE, Flynn JE (1975). Primary and secondary repair of flexor and extensor tendon injuries. *Hand Surgery*.

[7] El-Gammal TA, Steyers CM, Blair WF, Maynard JA (1993). Anatomy of the oblique retinacular ligament of the index finger. *The Journal of Hand Surgery*.

[8] Abrams RA, Ziets RJ, Lieber RL, Botte MJ (1997). Anatomy of the radial nerve motor branches in the forearm. *The Journal of Hand Surgery*.

[9] von Schroeder HP, Botte MJ, Gellman H (1990). Anatomy of the juncturae tendinum of the hand. *The Journal of Hand Surgery*.

[10] Botte MJ, Gelberman RH, Smith DG (1987). Repair of severe muscle belly lacerations using a tendon graft. *The Journal of Hand Surgery*.

[11] Hanz KR, Saint-Cyr M, Semmler MJ, Rohrich RJ (2008). Extensor tendon injuries: acute management and secondary reconstruction. *Plastic and Reconstructive Surgery*.

[12] Rockwell WB, Butler PN, Byrne BA (2000). Extensor tendon: anatomy, injury, and reconstruction. *Plastic and Reconstructive Surgery*.

